# Noise regulation by quorum sensing in low mRNA copy number systems

**DOI:** 10.1186/1752-0509-5-11

**Published:** 2011-01-20

**Authors:** Marc Weber, Javier Buceta

**Affiliations:** 1 Computer Simulation and Modelling (Co.S.Mo.) Lab, Parc Científic de Barcelona, C/Baldiri Reixac 10-12, Barcelona 08028, Spain

## Abstract

**Background:**

Cells must face the ubiquitous presence of noise at the level of signaling molecules. The latter constitutes a major challenge for the regulation of cellular functions including communication processes. In the context of prokaryotic communication, the so-called quorum sensing (QS) mechanism relies on small diffusive molecules that are produced and detected by cells. This poses the intriguing question of how bacteria cope with the fluctuations for setting up a reliable information exchange.

**Results:**

We present a stochastic model of gene expression that accounts for the main biochemical processes that describe the QS mechanism close to its activation threshold. Within that framework we study, both numerically and analytically, the role that diffusion plays in the regulation of the dynamics and the fluctuations of signaling molecules. In addition, we unveil the contribution of different sources of noise, intrinsic and transcriptional, in the QS mechanism.

**Conclusions:**

The interplay between noisy sources and the communication process produces a repertoire of dynamics that depends on the diffusion rate. Importantly, the total noise shows a non-monotonic behavior as a function of the diffusion rate. QS systems seems to avoid values of the diffusion that maximize the total noise. These results point towards the direction that bacteria have adapted their communication mechanisms in order to improve the signal-to-noise ratio.

## Background

Gene regulation at the transcriptional level is one of the corner stones of molecular and cellular biology [1]. Recent studies in prokaryotes have revealed the existence of antisense and alternative transcripts and multiple regulators per gene that imply a highly dynamic transcriptome more similar to that of eukaryotes than first thought [2]. Still, prokaryotic gene regulation mainly relies on the binding of regulatory proteins that attach to DNA for either stimulating or repressing transcription. These binding/unbinding events are intrinsically probabilistic because of the significance of thermal fluctuations at that scale and the low number of molecules involved in the process. In this regard, over the past years a growing number of experiments have indeed characterized not only the levels of randomness in cellular biochemical processes but also their functionality [3-8].

Technical advances such as the use of fluorescent tags in single-cell experiments have allowed for quantitative measurements of the noise in protein concentration and have shed light on the mechanisms of gene expression that lead to cell-to-cell variability [9-11]. Moreover, the advent of experimental approaches that permit to count individual mRNA and protein molecules in single cells has further evidenced the role played by fluctuations and their characteristics [12,13]. Thus, in *E. coli*, the direct measurement of integer-valued numbers of mRNA as a function of time has revealed transcriptional bursts with Poissonian statistics [14]. The latter is in agreement with the two-state gene expression model where switching between the active and inactive transcriptional regimes occurs with constant probability [15]. It is worth noticing that these noisy sources, far for being a nuisance, have been recognized to play a constructive role in many gene regulatory processes. Examples in this direction include the efficiency of the phage lambda switch [16] or the differentiation into the competence state in bacteria [17]. All in all, it is now accepted that stochastic and non-linear approaches are required for understanding the randomness in the dynamics of biochemical reactions and the effects of fluctuations in gene regulatory networks [7,8].

While a lot of modeling studies have focused on the single cell level [18,19], few of them have addressed the role played by noise at the colony level [20-23]. Our recent contributions within this topic, that illustrate the constructive role of stochasticity, include the noise-induced coherence resonance phenomenon in multicellular circadian clocks [24] and the interplay between the stochasticity of the cell cycle duration and the protein expression noise in bacterial colonies [25]. One relevant question within this context is how cellular populations deal with stochasticity in communication processes. In particular, we focus on the simplest cell-to-cell communication mechanism in prokaryotes: the so-called *quorum sensing *(QS). The term QS generically refers to the mechanism that allow bacteria to count their number, i.e. the colony size, by producing, exporting/importing into/from the environment, and detecting a diffusive signaling molecule, namely, the autoinducer [26,27]. As the population of bacteria grows, the autoinducer accumulates both in the extracellular medium and inside the cells. When the autoinducer concentration surpasses a threshold the expression of QS-controlled genes starts. This mechanism ultimately results in a response of the colony in a cell density dependent manner. Importantly, QS has opened the door to the design of gene circuits using synthetic biology approaches that control cell populations at the collective level [28-31].

Recent studies have shown that diffusion in QS reduces the noise at the level of the autoinducer [23]. However, to the best of our knowledge, the role played by different sources of stochasticity and their contribution to the dynamics of the signaling molecule has not been characterized yet. Moreover, while in eukaryotes the diffusion seems to contribute for enhancing the precision of regulatory processes, similar effects have not been reported in the context of QS [32]. We point out that a deep understanding of these issues is key in order to design robust synthetic circuits based on such bacterial communication system. Herein, we address these problems by studying the interplay between the QS communication and the transcriptional noise in bacterial populations. First, we aim at understanding how that interaction determines the dynamics of the autoinducer. Second, we aim at shedding light on the mechanisms that confer robustness to noise in QS communication. Transcriptional noise is expected to be particularly relevant when transcription events are short and rare. Under these conditions two main sources of stochasticity naturally arise: the dichotomous fluctuating dynamics of mRNA and the intrinsic noise due to low copy number of species. Thus, we restrict ourselves to the study of the aforementioned problems near the QS activation threshold where we can assume that the transcription events produce basal constitutive levels of mRNA as low as one molecule per cell at a time. We note that such mRNA production processes have been experimentally validated in prokaryotes revealing that the statistics of proteins bursts originates from the translation of a single mRNA molecule [33].

Our main findings are threefold. First, we show how the diffusion process leads to a repertoire of dynamics in regards of the signaling molecule. Second, we demonstrate that, for a large range of diffusion rate values, the main contribution to the total noise of the autoinducer concentration is the mRNA fluctuations. Finally, we show that the total noise exhibits a non-monotomic behavior as a function of the diffusion rate in contrast to previous results [23].

The paper is organized as follows. In the Methods section we introduce our modeling approach, the analytical calculations, and the parameter values used in our *in silico *experiments. In the next section, Results, we present our findings and compare the results from stochastic simulations with those from analytical calculations. Finally, we discuss further implications of our results in the Discussion, and summarize our findings in the Conclusion.

## Methods

### Modeling Approach

A large class of gram-negative bacteria use acyl homoserine lactones (AHLs) as signaling molecules [27,34]. These autoinducer molecules are typically synthesized by enzymes of the LuxI family and can freely diffuse across the cell membrane, i.e. by means of passive diffusion. When the concentration of autoinducer surpasses a critical threshold, it binds to its receptor (a cytoplasmic protein of the LuxR family) which then activates the expression of target genes, e.g. in Vibrio fisheri species the luxICDABE operon, that is responsible for the expression of luxI and luciferase. In contrast, when the autoinducer concentration is below the activation threshold, the transcription of the luxI gene occurs at a low basal rate, thus producing low levels of the enzyme. In this regime, the feedback regulation of the luxI gene leading to autoinduction can be disregarded. As a matter of fact, a number of QS systems lack autoinduction [35]. Consequently, to neglect the feedback in those cases is a valid approximation even above the activation threshold. Herein, for the sake of simplicity, we focus on this situation and describe the dynamics of the autoinducer near, but below, the activation threshold when feedback loops can be neglected and the downstream QS genes are not activated. We stress that, generally speaking, our results are not applicable above the activation threshold since the dynamics of the autoinducer is obviously conditioned by feedback effects.

Following previous approaches we consider a two-stage model for gene expression/regulation [15,36-38]. Thus, we assume that during the transcription events a single mRNA molecule, e.g. a luxI transcript, is produced and its dynamics can be then described by means of a Markovian dichotomous process [39],

(1) $$M_{0}^{i}\overset{\beta }{\underset{\alpha }{⇄}}M_{1}^{i}$$

where $M_{0,1}^{i}=0,1$ stands for the number of mRNA molecules at cell *i *and *α *and *β *for the transition rates between these states; i.e. *α *and *β *account for the probabilities per unit of time of mRNA degradation and transcription frequency respectively. Notice that the stochastic alternation of the mRNA between the values 0 (no mRNA) and 1 (a mRNA molecule) is not memoryless, i.e. white. Once a mRNA molecule is produced, and until it becomes degraded, the cell keeps noticing its presence and keep producing the autoinducer. That is, the transcriptional noise is a colored noise, and its autocorrelation decays exponentially with a characteristic time scale *τ_c _*= (*α *+ *β*)^-1 ^[39].

Once a mRNA molecule is produced the translational, and post-translational processes (if any), leads to the appearance of functional LuxI synthetases. Yet, our interest here focuses on the dynamics of the signaling molecule. It has been shown that the amount of the synthetase substrate is not a limiting factor for the production of the autoinducer [40,41]. As a consequence, the levels of the signaling molecule depends directly on the expression levels of the synthetase. Ignoring intermediate biochemical steps in the autoinducer synthesis reduces the number of noise sources and may even change, under some circumstances, the observed dynamics [42]. Still, it is a valid approximation in many situations, and here we assume that the translation of the synthetase and the subsequent synthesis of the autoinducer, *A*, can be effectively described by a single chemical step with rate k_+_. In addition, we consider that the autoinducer becomes degraded at a rate *k*_-_, that is,

(2) $$M_{1}^{i}\overset{k_{+}}{\to }M_{1}^{i}+A^{i}$$

(3) $$A^{i}\overset{k_{-}}{\to }\;\;\emptyset$$

Passive diffusion of the autoinducer can be implemented by considering a new species, *A*_ext_, that accounts for the number of signaling molecules in the extracellular medium such that,

(4) $$A^{i}\overset{D}{\underset{rD}{⇄}}A_{\text{ext}}$$

where *D *stands for the diffusion rate and *r *= *V*/*V*_ext _represents the ratio of the volume of a cell to the total extracellular volume. We consider all cells to have the same value of the diffusion rate. In addition, we assume a well-stirred system where spatial effects can be neglected.

As the bacterial population grows the autoinducer accumulates in the media. In experiments, in order to keep the concentration of the autoinducer below the activation threshold, such growth is compensated by means of a dilution protocol. As detailed below (see Parameters) the latter constitutes the main source of effective degradation of the signaling molecule. Thus, hereinafter we assume the degradation rate of the signaling molecule to be the same inside and outside the cell,

(5) $$A_{\text{ext}}\overset{k_{-}}{\to }\;\;\emptyset$$

**Figure 1:**
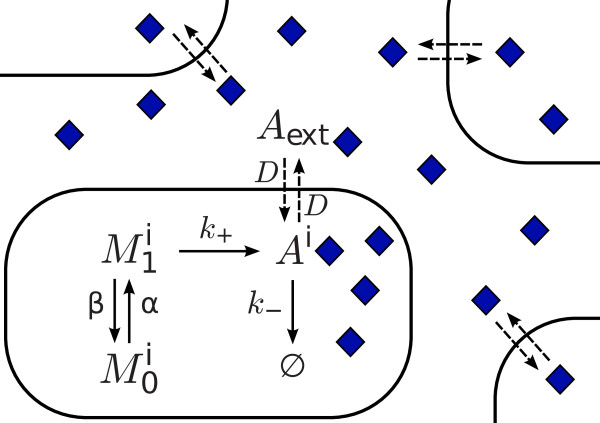
**Scheme of a simplified biochemical network of QS systems near the activation threshold**. Schematic representation of the biochemical processes considered in our approach for describing the dynamics of the signaling molecule, *A*, in cell *i*. The mRNA dynamics satisfies a dichotomous process characterized by the states *M*_0,1 _corresponding to zero and one molecules respectively. Once the autoinducer has been produced, it can diffuse in and out the cell leading to cell communication (see text).

Figure 1 schematically represents the biochemical processes considered in our approach. The set of reactions (1)-(5) characterizes the stochastic dynamics of the autoinducer and that of the mRNA. Their probabilistic description is given by the corresponding master equation that is *exactly *sampled by means of the Gillespie algorithm in a *N*-cells system [43].

### Analytical Calculations: Null Intrinsic Noise Approximation

Further insight into the dynamics of the signaling molecule can be obtained by analytical means as follows. Two stochastic contributions drive the dynamics of *A*: the mRNA fluctuations due to the random switching (mRNA present or not) and the molecular, i.e. intrinsic, noise due to low copy number of the autoinducer. As for the latter, it can be neglected if over the course of time *A^i^*/(*A^i ^*+ 1) ≃ 1 (large number of autoinducer molecules). While in our system this approximation is not totally justified (see parameters values below), it is useful to implement it in order to discriminate between the effects caused by different stochastic contributions and to obtain analytical expressions. In this case, it is straightforward to demonstrate that the dynamics of the autoinducer, Eqs. (1)-(5), can be described by the following coupled stochastic equations,

(6) $${\dot{c}}_{A^{i}}=k_{+}c_{M_{1}^{i}}(t)-k_{-}c_{A^{i}}+D(c_{A_{\text{ext}}}-c_{A^{i}})$$

(7) $$\begin{array}{c} {\dot{c}}_{A_{\text{ext}}}=-k_{-}c_{A_{\text{ext}}}+rD\sum_{i=1}^{N}(c_{A^{i}}-c_{A_{\text{ext}}})\\ =-k_{-}c_{A_{\text{ext}}}+rDN(\langle c_{A}\rangle -c_{A_{\text{ext}}}), \end{array}$$

where $c_{A^{i}}=A^{i}/V$, $c_{M_{1}^{i}}\left(t\right)=M_{1}^{i}/V,$ and $c_{A_{\text{ext}}}=A_{\text{ext}}/V_{\text{ext}}$ stand for the concentration of species *A *and $M_{1}^{i}$ at cell *i *and for species *A*_ext _at the extracellular medium respectively, *N *is the colony size (number of cells), and 〈·〉 represents the population average. In Eq. (6) the term $c_{M_{1}^{i}}\left(t\right)$ accounts for a dichotomous stochastic process characterized by the rates and states (*α*, *β*) and (0, *V *^−1^) respectively, and describes the fluctuating mRNA dynamics. We point out that in case that *D *= 0, Eq. (6) has been proposed to study graded and binary responses in stochastic gene expression. Interestingly, it has been shown that despite its simplicity it can actually reproduce some gene expression phenomena [37,44].

We can further proceed with the analytical calculations by implementing, as in previous studies e.g. [45], a quasisteady approximation for the dynamics of the external autoinducer, i.e. ${\dot{c}}_{A_{\text{ext}}}=0,$ so that,

(8) $$c_{A_{\text{ext}}}=\langle c_{A}\rangle \frac{1}{1+\frac{k_{-}}{NDr}}.$$

By substituting Eq. (8) into Eq. (6) we obtain an equation for the concentration of the signaling molecule inside a given cell that depends on the average 〈*c_A_*〉(the index *i *has been dropped),

(9) $$\begin{array}{c} {\dot{c}}_{A}=k_{+}c_{M_{1}}\left(t\right)-D\left(1+\frac{k_{-}}{D}\right)c_{A}\\ +\langle c_{A}\rangle \frac{D}{1+\frac{k_{-}}{N\;Dr}} \end{array}$$

In the absence of diffusion, Eq. (9) reveals that the concentration of the signaling molecule reaches a maximum value of $c_{A}^{+}=k_{+}/\left(k_{-}V\right)$ when $c_{M_{1}}(t)=V^{-1}$. In terms of $c_{A}^{+}$ and the time scale *t_c _*= 1/*k*_-_, the typical lifetime of a signaling molecule, the dimensionless version of Eq. (9) reads

(10) $${\dot{\tilde{c}}}_{A}={\hat{c}}_{M_{1}}\left(\tilde{t}\right)+k_{+}^{\text{eff}}\left(\langle {\tilde{c}}_{A}\rangle \right)-k_{-}^{\text{eff}}{\tilde{c}}_{A},$$

where $\tilde{D}=D/k_{-},k_{\_}^{\text{eff}}=1+\tilde{D},$ and $k_{+}^{\text{eff}}(\langle {\tilde{c}}_{A}\rangle )=\langle {\tilde{c}}_{A}\rangle \tilde{D}/\left(1+\left(1/N\tilde{D}r\right)\right);\;{\hat{c}}_{M_{1}}(\tilde{t})$ being a Markovian dichotomous noise characterized by the states $\left\{{\hat{c}}_{M_{1}}\right\}=0,1$ and the rates $\tilde{\alpha }=\alpha /k_{-}$ and $\tilde{\beta }=\beta /k_{-}$ Equation (10) can be formally closed by invoking the following self-consistency condition:

(11) $$\langle {\tilde{c}}_{A}\rangle =\int_{\tilde{\Omega }}{\tilde{c}}_{A\rho }({\tilde{c}}_{A};\langle {\tilde{c}}_{A}\rangle )\;d{\tilde{c}}_{A},$$

$\rho ({\tilde{c}}_{A};\langle {\tilde{c}}_{A}\rangle )$ being the probability density solving Eq. (10) and $\tilde{\Omega }$ its support [39]:

(12) $$\begin{array}{c} \rho ({\tilde{c}}_{A};\langle {\tilde{c}}_{A}\rangle )=N{\left(k_{-}^{\text{eff}}{\tilde{c}}_{A}-k_{+}^{\text{eff}}(\langle {\tilde{c}}_{A}\rangle )\right)}^{\frac{\tilde{\beta }}{k_{-}^{\text{eff}}}\;-\;1}\\ .{\left(1+k_{+}^{\text{eff}}(\langle {\tilde{c}}_{A}\rangle )-k_{-}^{\text{eff}}{\tilde{c}}_{A}\right)}^{\frac{\tilde{\alpha }}{k_{-}^{\text{eff}}}\;-\;1} \end{array}$$

with

(13) $$N=\frac{(1+\tilde{D})\Gamma \left[\frac{\tilde{\alpha }+\tilde{\beta }}{1+\tilde{D}}\right]}{\Gamma \left[\frac{\tilde{\alpha }}{1+\tilde{D}}\right]\Gamma \left[\frac{\tilde{\beta }}{1+\tilde{D}}\right]}$$

being the normalization constant. The condition (11) can be exactly solved and leads to the following value for the average concentration:

(14) $$\begin{array}{c} \langle {\tilde{c}}_{A}\rangle =\frac{1+\tilde{D}Nr}{1+\tilde{D}Nr+\tilde{D}}\frac{\tilde{\beta }}{\tilde{\alpha }+\tilde{\beta }}\\ =\frac{1+\tilde{D}Nr}{1+\tilde{D}Nr+\tilde{D}}\langle {\tilde{c}}_{A}\rangle {|}_{\tilde{D}=0} \end{array}$$

where $\langle {\tilde{c}}_{A}\rangle {|}_{\tilde{D}=0}=\tilde{\beta }/\left(\tilde{\alpha }+\tilde{\beta }\right)$ is the average concentration of signaling molecules in the absence of diffusion. Thus, as expected, $\langle {\tilde{c}}_{A}\rangle <\langle {\tilde{c}}_{A}\rangle {|}_{\tilde{D}=0}$. For the sake of concision, on what follows we drop the argument term $\langle {\tilde{c}}_{A}\rangle$ from the notation of $\rho ({\tilde{c}}_{A};\langle {\tilde{c}}_{A}\rangle )$. Note that $\rho ({\tilde{c}}_{A})$ has two states (barriers) that define its support. That is, the minimum and maximum values that the concentration of the autoinducer can reach as a function of the diffusion are:

(15) $$c_{A}^{-}=\frac{{\tilde{D}}^{2}Nr}{\left(1+\tilde{D})(1+\tilde{D}+\tilde{D}Nr\right)}\frac{\tilde{\beta }}{\tilde{\alpha }+\tilde{\beta }}$$

(16) $${\tilde{c}}_{A}^{+}=\;\;{\tilde{c}}_{A}^{-}+\frac{1}{1+\tilde{D}}$$

It is easy to prove that the probability density $\rho ({\tilde{c}}_{A})$ shows a single extremum if,

(17) $$\tilde{\alpha },\tilde{\beta }≶k_{-}^{\text{eff}},$$

**Figure 2:**
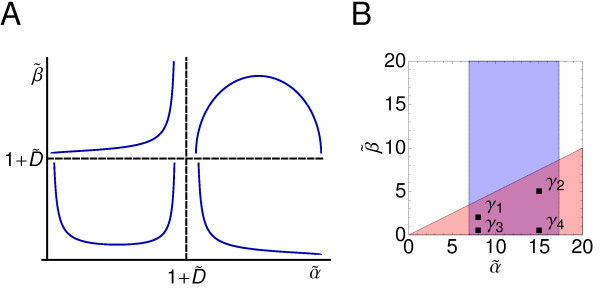
**Probability densities of the signaling molecule and parameter space**. Panel **A**: Schematic representation of the different probability densities of the autoinducer concentration depending on the value of $\tilde{\alpha }$ and $\tilde{\beta }$ with respect to that of $\tilde{D}$. Given a set of values ($\tilde{\alpha }$, $\tilde{\beta }$) the dynamics of the autoinducer shows different behaviors depending on the value of the diffusion parameter since the transitions lines are located at $\tilde{\alpha }$, $\tilde{\beta }$ = 1 + $\tilde{D}$. The constraints of our modelling in terms of the parameters values make the region on the top-left corner non-accessible (see text). Panel **B**: Parameter space diagram ($\tilde{\alpha }$, $\tilde{\beta }$) indicating the sets of parameters used in the simulations (solid squares): γ_1 _= (8, 2), γ_2 _= (15, 5), γ_3 _= (8, 0.5), γ_4 _= (15, 0.5). The experimental values reported for the degradation rate of the mRNA leads to a biological meaningful range for $\tilde{\alpha }$ (blue region). The low constitutive expression assumption is prescribed by the constraint $\tilde{\alpha }>2\tilde{\beta }$ (red colored region).

Where the extremum is a maximum if $\tilde{\alpha },\tilde{\beta }>k_{-}^{\text{eff}}$ and a minimum if $\tilde{\alpha },\tilde{\beta }<k_{-}^{\text{eff}}$. In the other cases the probability density does not display any extrema. Therefore, as a function of $\tilde{\alpha }$ and $\tilde{\beta }$, the probability density $\rho ({\tilde{c}}_{A})$ may show four different behaviours depending on the value of the diffusion coefficient as schematically represented in Figure 2A. However, a constraint in our modeling restricts the regions, i.e. behaviors, accessible to the autoinducer dynamics. We have assumed a low constitutive expression such that only a single mRNA molecule can be transcribed at a time. The latter implies that $\tilde{\beta }<\tilde{\alpha }$

(the degradation rate of the mRNA is larger than the transcription rate) in order to assure that a maximum of one mRNA molecule is present in a cell at a given time. As a consequence, and independently of the diffusion value, the dynamics leading to the probability density shown at the top-left region of Figure 2A cannot be considered as physical in the context of our modeling approach. We stress that this constraint is not a fundamental ingredient for obtaining our results (see Discussion).

Finally, the noise of the autoinducer concentration reads,

(18) $$\begin{array}{c} \eta_{{\tilde{c}}_{A}}^{2}=\frac{\sigma_{{\tilde{c}}_{A}}^{2}}{{\langle {\tilde{c}}_{A}\rangle }^{2}}\\ =\frac{\tilde{\alpha }{\left(1+\tilde{D}+\tilde{D}Nr\right)}^{2}}{\beta \left(1+\tilde{D}\right){\left(1+\tilde{D}Nr\right)}^{2}\left(1+\tilde{D}+\tilde{\alpha }+\tilde{\beta }\right)} \end{array}$$

where $\sigma_{{\tilde{c}}_{A}}^{2}=\langle {\tilde{c}}_{A}^{2}\rangle -{\langle {\tilde{c}}_{A}\rangle }^{2}$.

Nonetheless we have disregarded the intrinsic noise in the analytical calculations, Eq. (18) will allow us to elucidate the contributions of different sources of noise as follows. By means of the numerical simulations (Gillespie) of the set of reactions (1)-(5) in a *N*-cell system, we can evaluate the total (intrinsic+transcriptional) noise. Hence, by substracting from that quantity the contribution of the transcriptional noise, i.e. Eq. (18), we obtain the levels of intrinsic noise (see Results).

### Parameters

We are interested in the role played by the fluctuations of the signaling molecule, *A*, when its concentration is close to the activation of the QS switch. Therefore, we fix the mean concentration of the autoinducer and modulate the rest of the parameters in order to keep constant this value. Pai and You [46] have recently studied the core architecture of the QS mechanism for a comprehensive set of systems. These authors have estimated that the critical concentration of autoinducer needed for the activation of the QS genes ranges from 10 to 50 nM for most of the bacterial species. In our model, we set the average concentration of *A *to a typical value of $C_{A}^{0}$ = 25 nM. Yet, our results do not depend on the particular value we choose within that range. As shown below, see Results, this value fixes the level of intrinsic noise of the system. However, the interplay between diffusion and transcriptional noise does not depend on that. Moreover, by defining the so-called sensing potential, *ν *= (*rN*)^-1 ^Pai and You estimated the range of critical cell densities for the QS activation. They concluded that its characteristic value is *ν *~ 10^3 ^- 10^4^. In our simulations we set this parameter to *ν *- 10^3^. In the experimental setups the cells are typically present in a volume of a few milliliters and the total number of cells is of the order of 10^8 ^- 10^9^. Therefore, the concentration of autoinducer in the medium is determined by the exchange of signaling molecules coming from many cells. In contrast, the behavior of QS systems with a very low number of cells can be significantly different, as shown by microfluidic confinement of cells in picoliter droplets [47-49]. In our study, in order to discard small system size effects, we choose a sufficiently large number of cells in the numerical simulations, *N *= 10^2^. Since the typical volume of an *E*. *coli *cell is *V *= 1.5 *μ*m^3 ^then *V*_ext _= 10^5^*V *(i.e. *r *= 10^−5^). We point out that keeping *ν *to a constant value necessarily requires an external dilution protocol for maintaining constant the cell density. In experiments, the control of the dilution rate is usually achieved by the use of chemostats or microfluidic devices [50]. The rate of dilution should compensate for the cell growth, ~ 2 · 10^−2 ^min^−1 ^(i.e. cell cycle duration ~50 min). In our modeling, by keeping constant the number of cells and the average concentration of the autoinducer, we tacitly assume a dilution protocol too. Importantly, the dilution rate effectively modifies the degradation rate of the signaling molecule. In this regard, while some bacteria species have hydrolytic enzymes that degrade AHLs, generally speaking, bacteria that synthetize AHLs do not degrade them enzymatically. In fact, AHLs are chemically stable species in aqueous solutions [51]. The degradation rate of the homoserine lactone 3-Oxo-C6-AHL has been measured *in vitro *revealing that this autoinducer is rather stable: ~ 3 · 10^−4 ^min^−1 ^[51]. Measurements of the degradation rate of other AHL autoinducers show similar results. Based on experimental data and mathematical modeling, the degradation rate of the signaling molecule *in vivo *has been also estimated [46]. Depending on the pH of the medium, the latter ranges from ~ 5 · 10^−3 ^min^−1 ^to ~ 2 · 10^−2 ^min^−1^. Consequently, the dilution process constitutes the main source of effective degradation of *A*, both inside and outside the cell, and here we set *k*_− _= 2 · 10^−2 ^min^−1^.

As for the value of the diffusion rate, the coefficient of passive diffusion has been estimated for the 3-Oxo-C6-AHL autoinducer based on the measure of the diffusion of glucose and lactose through the outer membrane of *E. coli *[23]. For a typical cell volume of 1.5 *μ*m^3 ^the estimated coefficient of diffusion is ~ 10^3 ^min^−1^. Under these conditions the typical value for the dimensionless parameter $\tilde{D}$ is of the order of 10^4^. Yet, active transport mechanisms for the autoinducer leads to much smaller effective diffusion values (see Discussion) and we explore the role played by this parameter.

In regards to the mRNA dynamics, *α*, the degradation rate, depends on the cell degradative machinery. To this respect, the half-lives of all mRNAs of *Staphylococcus aureus *have been recently measured during the mid-exponential phase. Most of the transcripts, 90%, have half-lifes shorter than 5 minutes [52,53].

According to these studies we restrict the mRNA degradation rate to the range ln(2)/5 min^−1 ^< α < ln(2)/2 min^−1^. Consequently, $\tilde{\alpha }$ > 1. As for the frequency of the transcription events, *β *is determined by particular characteristics of the gene regulatory process under consideration, e.g. the affinity of the regulatory proteins to the operator site and the initiation rate of transcription. Due to the assumption of a low constitutive expression, we choose values of parameter *β *satisfying the relation *α *>*β*. In particular, we implement the more restrictive condition *α *> 2*β*. Figure 2B recapitulate these constraints and show the different sets of $\tilde{\alpha }$ and $\tilde{\beta }$ values that we have used in our simulations and analytical calculations.

Summarizing, *N*, *r*, and *k*_− _are kept fixed in our simulations and analytical calculations and we explore the parameter space *α*, *β*, and *D *within the ranges, and satisfying the constraints, mentioned above. In every particular situation, once a set of those parameters is prescribed, we set the value of *k*_+ _by using Eq. (14) in order to keep the average value of *c_A _*near its critical concentration value (25 nM).

## Results

### Comprehensive Study of the Autoinducer Dynamics as a Function of the Diffusion Rate

**Figure 3:**
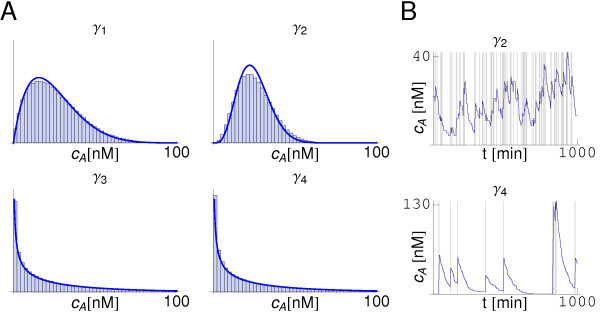
**Distributions and dynamics of the signaling molecule in a diffusionless system**. Panel **A**: Distributions of *c_A _*at steady-state for different sets of parameters ($\tilde{\alpha }$, $\tilde{\beta }$) as indicated in Figure 2B. In all cases $\tilde{D}$ = 0. The histogram obtained in simulations (blue bars) compares well with the distribution from the analytical calculations (blue line). Yet, deviations are observed due to intrinsic noise (see text). Panel **B**: The dynamics of the autoinducer show different behaviors depending on the region of the parameters phase space (see Figure 2). Two typical trajectories are shown with a grey-shaded background indicating the presence of a mRNA molecule in the cell.

The distribution of *c_A _*at the steady-state is computed for the different parameter sets according to the ranges and constraints described above (section Methods). In order to explore the role of the diffusion in the dynamics of the signaling molecule we first study the case $\tilde{D}=0$. According to the analytical calculations, see Eq. (17), in this case two possible distributions for the concentration of *c_A _*can be observed depending on the value of $\tilde{\beta }$. Since $\tilde{\alpha }$ > 1 we can expect a maximum only if $\tilde{\beta }$ > 1 (note that $k_{-}^{\text{eff}}=1$ if $\tilde{D}=0$), otherwise extrema are not expected. The results of the numerical simulations (Gillespie), Figure 3A, reveal that scenario. Note that in all cases the histogram obtained from the simulations fits fairly well to the expression (12) except for deviations due to the intrinsic noise that are not taken into account by the analytical approach. The differences among dynamics are evidenced by the trajectories, Figure 3B. Thus, for $\tilde{\beta }$ < 1 the dynamics of the autoinducer shows a burst-like behavior. If $\tilde{\beta }$ > 1 the frequency of bursts is high enough to maintain the concentration of signaling molecules near the average and a single-peak distribution develops.

**Figure 4:**
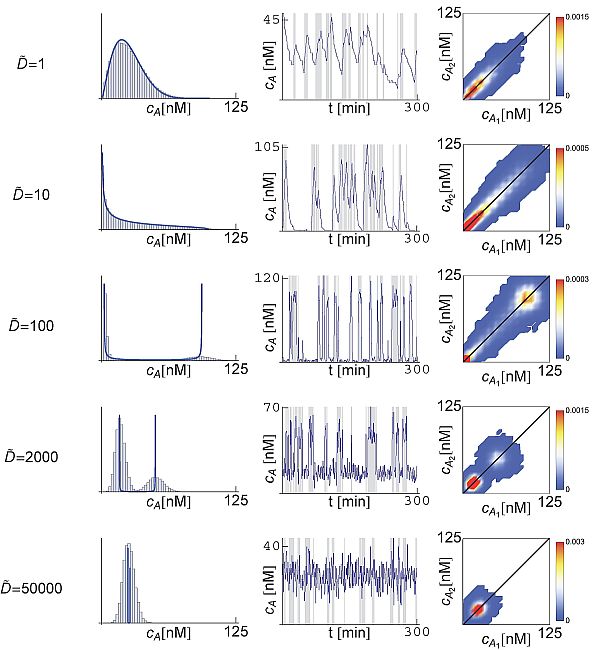
**Distributions and dynamics of the signaling molecule in a system with diffusion**. Distributions (left column) and dynamics (center column) of *c_A _*at steady-state for different values of $\tilde{D}$. The right most column stands for a density plot of the distribution of $c_{A_{1}}$ as a function of $c_{A_{2}}$ for discerning a putative increase in the molecular noise (see text). In all cases the parameters set ($\tilde{\alpha }$, $\tilde{\beta }$) is γ_2 _(see Figure 2B). The production rate ${\tilde{k}}_{+}$ is modulated as a function of ($\tilde{\alpha }$, $\tilde{\beta }$, $\tilde{D}$) in order to maintain constant the average 〈*c_A_*〉 = 25 nM. The histograms obtained in the stochastic simulations (blue bars, left column) are in qualitative agreement with the probability densities from the analytical calculations (blue line, left column). When increasing the diffusion coefficient, the system explores different dynamics as revealed by the trajectories shown in the center column. The grey-shaded background shown in the trajectories of *c_A _*indicates the presence of a mRNA molecule in the cell. The density plots (right column) reveals that the diffusion does not contribute to an increase of the intrinsic noise since the spreading of the distributions in a direction perpendicular to the diagonal does not grow.

If $\tilde{D}$ > 0 we expect a more fruitful phenomenology since the transition lines between behaviors in the parameter space ($\tilde{\alpha }$, $\tilde{\beta }$) shift as a function of the diffusion (see Figure 2A). According to the analytical calculations we can anticipate that, for a given parameter set and as $\tilde{D}$ increases, the system explores different dynamical regimes. By taking as a reference the case γ_2_, that is ($\tilde{\alpha }$, $\tilde{\beta }$) = (15, 5), Figure 4 shows the effect of the diffusion on the distribution (left column) and dynamics (center column) of *c_A _*in a given cell. The system initially displays a single-peak distribution for $\tilde{D}$ = 1. By increasing the diffusion coefficient we observe transitions to the other phases (monotonically decreasing and double-peak distributions). The corresponding dynamics of *c_A _*(right panels) show how the diffusion, acting as an additional effective degradation on *A*, first increases the sharpness of the bursts of production. For $\tilde{D}$ = 10, the diffusion is large enough to remove signaling molecules between consecutive burst events, thus leading to a monotonically decreasing distribution. Increasing the diffusion rate to $\tilde{D}$ = 100 leads to the situation where both $\tilde{\alpha }$ and $\tilde{\beta }$ becomes smaller than 1 + $\tilde{D}$ and a bistable dynamics develops. Under these circumstances the concentration of autoinducer alternates between two states that correspond to a low concentration, when there is no mRNA production, and a high concentration, following the mRNA synthesis. As the diffusion further increases, e.g. $\tilde{D}$ = 2 · 10^3^, the autoinducer molecules diffusing from the external medium into the cell set a constitutive level of this species. The latter explains the presence of *A *molecules in the cell even if no mRNA is produced. Finally, at very large values of $\tilde{D}$, e.g. $\tilde{D}$ = 5 · 10^4^, the low constitutive concentration of the autoinducer increases due to the influx of molecules when no mRNA is present whereas the concentration of *A *that is internally produced decreases due to the efflux of molecules. In this case, the whole *N*-cells system can be considered as a single volume with no diffusive barriers between cells. Thus, the burst events average out and, as a consequence, a single effective peak appears.

### Diffusion and Intrinsic Noise

Figure 4 shows that the theoretical distribution captures the essential features of the dynamics obtained in numerical simulations (Gillespie). The noticeable deviations are due to the intrinsic noise that are not considered in the theoretical analysis. Notice that as the diffusion increases those deviations seem to be larger. We stress that in our simulations we keep constant the average concentration of the autoinducer by modulating the effective production rate (see Eq. (14)). That is, as $\tilde{D}$ becomes larger, we increase the production rate *k*_+ _so that the average number of autoinducer molecules per cell remains the same. Consequently, the deviations between simulations and the theoretical analysis cannot be ascribed to a putative intracellular decrease of the number of *A *molecules (i.e. to an increase of intrinsic noise). Moreover, the deviations cannot be attributed either to a failure of the quasisteady approximation introduced in Eq. (7) because the larger the diffusion, the more accurate that approximation is. As indicated by equation (18), the transcriptional noise behaves as ${\tilde{D}}^{-1}$. Thus, for large values of the diffusion rate, the transcriptional noise level decreases. Therefore, we must conclude that the deviations between the theoretical and the numerical approaches become accentuated as the diffusion increases because there is a drop of the fluctuations related to the mRNA dynamics. This also indicates that for large enough diffusion rate, the intrinsic noise constitutes the main source of stochasticity.

In order to ensure that the intrinsic fluctuations are not actually increasing due to diffusion we perform the following *in silico *experiment. We consider a modification of our system such that a single mRNA molecule transcript leads to two autoinducer molecules that are considered to be distinguishable. The latter can be experimentally achieved by placing two consecutive copies of the encoding sequence of the autoinducer synthetase labeled with different fluorescent tags in the operon. Thus, we double the set of equations (2)-(5) in order to account for $A_{1}^{i}$ and $A_{2}^{i}$ molecules synthesis at cell *i *due to a single mRNA transcript $M_{1}^{i}$. Following [9], by plotting the distribution of $c_{A_{1}}$ as a function of $c_{A_{2}}$ a putative increase of the intrinsic fluctuations can be discerned by these means. Right column of Figure 4 displays the results in this regard. The width of the distribution in a direction perpendicular to the diagonal is a measure of the intrinsic fluctuations (see [9] for details). As shown, as the diffusion increases there is no amplification of this quantity.

### Diffusion and Total Noise

**Figure 5:**
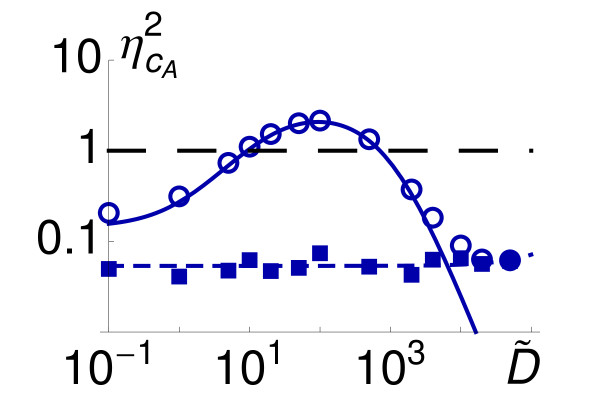
**Noise of the signaling molecule as a function of the diffusion coecient**. Noise $\eta_{c_{A}}^{2}$ as a function of diffusion coefficient $\tilde{D}$ for the set of parameters γ_2 _(see Figure 2B): stochastic simulations (circles) and analytical expression. Eq. (18), (solid line). By using the decomposition $\eta_{c_{A}}^{2}=\eta_{c_{A},\mathrm{int}}^{2}+\eta_{c_{A},\text{tran}}^{2}$ the differences between the computational and the theoretical distributions quantifies the amount of intrinsic noise (squares). As evidenced by the linear regression (blue short-dashed line) the later remains constant and is the main contribution to the total noise only for large diffusion values, $\tilde{D}$ > 10^4 ^(see text).

It is interesting to place the previous result in the context of the total noise of the autoinducer concentration. Figure 5 reveals that $\eta_{c_{A}}^{2}$ shows a non-monotonic behavior. As a function of $\tilde{D}$ the total noise first increases, reaches a maximum, and then decreases as the diffusion becomes larger. While Figure 5 represents data for the γ_2 _parameter set, this behavior applies for all the values of $\tilde{\alpha }$ and $\tilde{D}$ explored in our simulations (data not shown). We point out that if $\eta_{c_{A}}^{2}$ > 1 then the dispersion is larger than the mean. Under this circumstance the fluctuations can lead to catastrophic events. E.g., fluctuations are able to remove all the signaling molecules within the cell. Note that the analytical calculations, that just account for the transcriptional noise, are in agreement with the numerical simulations, that account for both the transcriptional and the intrinsic noise, for a large range of $\tilde{D}$ values. This indicates that the main contribution to the total fluctuations within a large range of diffusion values is the transcriptional noise. Yet, as mentioned above, the latter diminishes as the diffusion increases while the intrinsic fluctuations remain constant. Consequently, the contribution of the intrinsic noise must become more relevant than the mRNA stochasticity beyond some value of $\tilde{D}$.

We address this point quantitatively by calculating the relative importance of these noisy sources. To this end, we make use of the decomposition $\eta_{c_{A}}^{2}=\eta_{c_{A},\mathrm{int}}^{2}+\eta_{c_{A},\text{tran},}^{2}$ where $\eta_{c_{A},\mathrm{int}}^{2}$ and $\eta_{c_{A},\text{tran}}^{2}$, stand respectively for the intrinsic and the transcriptional contributions to the total noise [54]. Thus, by subtracting the analytical expression given by Eq. (18) to the total noise obtained in the numerical simulations, we are able to compute the intrinsic noise as a function of the diffusion. By performing a linear regression of the points that corresponds to the intrinsic noise we obtain that the slope of the curve is indeed zero in practical terms, ~ 2 · 10^−7^. Therefore, in agreement with the qualitative results obtained in Figure 4 (right column), the intrinsic noise remains constant as the diffusion increases, $\eta_{c_{A},\mathrm{int}}^{2}$ = 0.054 ± 0.003, and is the main stochastic component if $\tilde{D}$ > 10^4^.

## Discussion

As a matter of discussion, our modeling consider passive diffusion as the mechanism for the transport of the signaling molecule. This is indeed true in many QS systems. However, in other cases the autoinducer is actively transported in and out of the cell. For example, in the bacterial species *Pseudomonas aeruginosa*, C4-HSL can freely diffuse but C12-HSL, a larger signaling molecule, is subjected to active influx and efflux at rates of ~ 10^−2 ^min^−1 ^and ~ 10^−1 ^min^−1 ^respectively [55]. Other example corresponds to the AI-2 signaling molecule. The latter is present in many Gram-positive and Gram-negative species and it is believed to allow for interspecies communication [56]. In *E. coli *and *Salmonella enterica *extracellular AI-2 accumulates during the exponential phase, but then decreases drastically upon entry into the stationary phase. This reduction is due to the import and processing of AI-2 by the Lsr transporter [56,57]. Moreover, excretion from the cell of this autoinducer also appears to be an active process involving the putative transport protein YdgG (or alternatively named TqsA) [58]. In the case of *E. coli *these rates have been estimated by computational and experimental means: *D_out _*≃ 10^-1 ^min^−1 ^and *D_in _*≃ 10^-3 ^- 10^-2 ^min^−1 ^[59]. All in all, the diffusion rates when driven by active processes are four orders of magnitude smaller than the diffusion rates of small molecules through the membrane. Hence, the diffusion rates in QS systems can be categorized into two main, well separated, classes: small diffusion rates due to active transport, and large diffusion rates due to passive mechanisms.

In principle our model does not account for active diffusion processes, but transport driven by concentration differences. Still, it can be demonstrated that if the following condition holds, *r *≃ *D_in_*/*D_out_*, then our simple model fairly describes the active diffusion with *D *= *D_out_*. However, that regime is not accessible in our simulations near the critical sensing potential since that would imply a non-physical situation (*N *< 1). If in any case we consider the rates of active transport of these QS systems, they would fit in our model with a normalized effective diffusion coefficient in the range $\tilde{D}$ ∈ [10^-1^, 10]. Note that, either driven by passive diffusion or by active transport, the region that maximizes the total noise, $\eta_{c_{A}}^{2}$, is not accessible: $\tilde{D}$ ~ 5 · 10^1 ^− 10^2^. Notice also that this range of diffusion values corresponds to the dynamics that produce separated peaks in the distribution of the autoinducer (see Figure 4). While our modeling is certainly very simple and the derived consequences should be carefully taken, this observation suggest that bacteria have developed mechanisms for coping with the noise and keep their functional QS regime away from the region where $\eta_{c_{A}}^{2}$ > 1. In this regard, let us point out that in every form of information exchange the precision is key. If the precision of the information is fuzzy then the related biological function lacks robustness. Thus, our results point towards the direction that bacteria have adapted their communication mechanisms in order to improve the signal to noise ratio.

Diffusion can lessen the effects of fluctuations, both in eukaryotes [32] and prokaryotes [23,60]. This behavior is certainly obtained in our model: if the diffusion rate is larger than a given amount, then the noise decreases as the diffusion increases. However, to the best of our knowledge, the reverse effect, i.e. the noise increases as the diffusion increases, had not been reported. These opposed behaviors are responsible of the non-monotonic comportment of the noise described herein. One may wonder the reason underlying this phenomenology. By manipulating Eq. (18), it is easy to demonstrate that a) the slope of the noise at $\tilde{D}$ = 0 is always positive and b) in the white noise limit, *τ_c _*→ 0, the slope becomes null and the transcriptional noise decreases monotonously as the diffusion increases. Consequently, the observed behavior is due to the colored character of the transcriptional noise, i.e. due to a competition of temporal scales: those of the diffusion and the noise correlation time. This result opens the possibility of finding a similar phenomenology in other systems subjected to relevant levels of transcriptional noise and diffusive signals. In addition, it points up the relevance of non-memoryless noisy sources in biological systems [11]. Moreover, it shows that our results do not depend on details as the precise number of transcripts (one in our case). As long as there are distinct transcriptional phases inside cells with characteristic time scales, that is, presence versus absence of transcripts, the observed phenomenology is, qualitatively, the same. Yet, those phases are prone to appear when the autoinducer is produced at constitutive levels and the number of transcripts is small. Herein we restrict ourselves to the case of one transcript simply because we are able to obtain analytical results in that situation.

## Conclusions

Herein we have explored the role played by cell-cell communication and transcriptional noise in QS systems near the activation threshold. Within this context, we have shown that the dynamics of the signaling molecule exhibits different behaviors depending on the diffusion coefficient. When increasing the rate of diffusion, the probability distribution of the autoinducer changes from single-peak distribution (sustained bursts dynamics), to monotonically decreasing distribution (bursts dynamics), to double-peak distribution (bistable dynamics), and finally to narrow single-peak distribution (diffusion-averaged dynamics).

In addition, we have shown that the mRNA dynamics plays a crucial role for regulating the total amount of molecular noise of the signaling molecules. Transcriptional noise is the main contribution to the total noise for a large range of diffusion values, $\tilde{D}$ < 10^4^. Only for very large values of the diffusion the intrinsic noise is the major source of stochasticity. Due to a competition of temporal scales, the total noise shows a non-monotonic behavior as a function of the diffusion rate. For large values of the diffusion coefficient, the total noise decreases as the diffusion rate increases. In this regard, our results are to be compared to previously reported noise reduction mechanisms, as for example in the case of bistable genetic switches coupled by QS communication [60]. On the other hand, when the diffusion rate is small enough compared to the characteristic rate of transcription events, the total noise increases as the diffusion becomes larger. The values of the diffusion rates in QS systems fall into two distinctive categories: either large values corresponding to passive transport mechanism or small values when an active transport mechanism applies. Surprisingly, these two QS classes avoid diffusion rates that maximize the total noise. According to these results, we conjecture that bacteria have engineered the communication mechanism for reducing the signal-to-noise ratio and produce a more reliable information exchange.

Our final comment refers to the possibility of considering other sources of stochasticity. Cell-to-cell variability and extrinsic noise have been proved to act as an important contribution in many cell processes [16,25,61,62]. In the context of the problem studied herein, we can envision that variability, either at the level of the mRNA dynamics or at the level of the diffusion rate, can effectively lead to significant changes in the reported phenomenology. In addition, by considering additional steps in the synthesis of the autoinducer, the levels of intrinsic noise would increase. Whether or not this extra level of fluctuations, coupled with feedback regulation, may generate new effects in the framework of QS is not known. Work in those directions is in progress.

## Authors' contributions

MW and JB contributed equally to construct the model, perform the analytical calculations and write the paper. Numerical simulations were carried out by MW. All authors read and approved the final manuscript.

## References

[B1] Elf J Li GW Xie XS Probing transcription factor dynamics at the single-molecule level in a living cell Science 2007 316 5828 1191 4 10.1126/science.1141967 17525339 PMC2853898

[B2] Güell M van Noort V Yus E Chen WH Leigh-Bell J Michalodimitrakis K Yamada T Arumugam M Doerks T Kühner S Rode M Suyama M Schmidt S Gavin AC Bork P Serrano L Transcriptome complexity in a genome-reduced bacterium Science (New York, NY) 2009 326 5957 1268 71 10.1126/science.1176951 19965477

[B3] Kæ rn M Elston T Blake W Collins J Stochasticity in gene expression: from theories to phenotypes Nature Reviews Genetics 2005 6 6 451 464 http://www.nature.com/nrg/journal/v6/n6/abs/nrg1615.html 15883588 10.1038/nrg1615

[B4] Süel GM Garcia-Ojalvo J Liberman LM Elowitz MB An excitable gene regulatory circuit induces transient cellular differentiation Nature 2006 440 7083 545 50 16554821 10.1038/nature04588

[B5] Raj A van Oudenaarden A Nature, nurture, or chance: stochastic gene expression and its consequences Cell 2008 135 2 216 26 10.1016/j.cell.2008.09.050 18957198 PMC3118044

[B6] Brock A Chang H Huang S Non-genetic heterogeneity - a mutation-independent driving force for the somatic evolution of tumours Nature reviews. Genetics 2009 10 5 336 42 10.1038/nrg2556 19337290

[B7] Ao P Global view of bionetwork dynamics: adaptive landscape J Genet Genomics 2009 36 2 63 73 10.1016/S1673-8527(08)60093-4 19232305 PMC3165055

[B8] Eldar A Elowitz MB Functional roles for noise in genetic circuits Nature 2010 467 7312 167 73 10.1038/nature09326 20829787 PMC4100692

[B9] Elowitz MB Levine AJ Siggia ED Swain PS Stochastic gene expression in a single cell Science (New York, NY) 2002 297 5584 1183 6 10.1126/science.1070919 12183631

[B10] Ozbudak EM Thattai M Kurtser I Grossman AD van Oudenaarden A Regulation of noise in the expression of a single gene Nature genetics 2002 31 69 73 10.1038/ng869 11967532

[B11] Rosenfeld N Young JW Alon U Swain PS Elowitz MB Gene regulation at the single-cell level Science (New York, NY) 2005 307 5717 1962 5 10.1126/science.1106914 15790856

[B12] Raj A van Oudenaarden A Single-molecule approaches to stochastic gene expression Annual review of biophysics 2009 38 255 70 10.1146/annurev.biophys.37.032807.125928 19416069 PMC3126657

[B13] Larson DR Singer RH Zenklusen D A single molecule view of gene expression Trends in cell biology 2009 19 11 630 7 10.1016/j.tcb.2009.08.008 19819144 PMC2783999

[B14] Golding I Paulsson J Zawilski SM Cox EC Real-time kinetics of gene activity in individual bacteria Cell 2005 123 6 1025 36 10.1016/j.cell.2005.09.031 16360033

[B15] Shahrezaei V Swain PS Analytical distributions for stochastic gene expression Proceedings of the National Academy of Sciences of the United States of America 2008 105 45 17256 61 10.1073/pnas.0803850105 18988743 PMC2582303

[B16] Zhu XM Yin L Hood L Ao P Robustness, stability and efficiency of phage lambda genetic switch: dynamical structure analysis J Bioinform Comput Biol 2004 2 4 785 817 10.1142/S0219720004000946 15617166

[B17] Cağatay T Turcotte M Elowitz MB Garcia-Ojalvo J Süel GM Architecture-dependent noise discriminates functionally analogous differentiation circuits Cell 2009 139 3 512 22 19853288 10.1016/j.cell.2009.07.046

[B18] Kepler T Elston T Stochasticity in transcriptional regulation: origins, consequences, and mathematical representations Biophysical Journal 2001 81 6 3116 3136 10.1016/S0006-3495(01)75949-8 11720979 PMC1301773

[B19] Wilkinson DJ Stochastic modelling for quantitative description of heterogeneous biological systems Nature reviews. Genetics 2009 10 2 122 33 10.1038/nrg2509 19139763

[B20] Garcia-Ojalvo J Elowitz M Strogatz S Modeling a synthetic multicellular clock: Repressilators coupled by quorum sensing Proceedings of the National Academy of Sciences of the United States of America 2004 101 30 10955 10.1073/pnas.0307095101 15256602 PMC503725

[B21] Goryachev AB Toh DJ Wee KB Zhang HB Zhang LH Lee T Transition to quorum sensing in an Agrobacterium population: A stochastic model PLoS computational biology 2005 1 4 e37 10.1371/journal.pcbi.0010037 16170413 PMC1214540

[B22] Zhou T Chen L Aihara K Molecular Communication through Stochastic Synchronization Induced by Extracellular Fluctuations Physical Review Letters 2005 95 17 2 5 10.1103/PhysRevLett.95.178103 16383875

[B23] Tanouchi Y Tu D Kim J You L Noise reduction by diffusional dissipation in a minimal quorum sensing motif PLoS computational biology 2008 4 8 e1000167 18769706 10.1371/journal.pcbi.1000167 PMC2507755

[B24] Ullner E Buceta J Díez-Noguera A García-Ojalvo J Noise-Induced Coherence in Multicellular Circadian Clocks Biophysical Journal 2009 96 May 3573 3581 10.1016/j.bpj.2009.02.031 19413962 PMC2711409

[B25] Canela-Xandri O Sagués F Buceta J Interplay between Intrinsic Noise and the Stochasticity of the Cell Cycle in Bacterial Colonies Biophysical journal 2010 98 11 10.1016/j.bpj.2010.02.045 20513389 PMC2877355

[B26] Bassler BL Losick R Bacterially speaking Cell 2006 125 2 237 46 10.1016/j.cell.2006.04.001 16630813

[B27] Ng WL Bassler BL Bacterial quorum-sensing network architectures Annual review of genetics 2009 43 197 222 10.1146/annurev-genet-102108-134304 19686078 PMC4313539

[B28] You L Cox RS Weiss R Arnold FH Programmed population control by cell-cell communication and regulated killing Nature 2004 428 6985 868 71 10.1038/nature02491 15064770

[B29] Basu S Gerchman Y Collins C Arnold F Weiss R A synthetic multicellular system for programmed pattern formation Nature 2005 434 7037 1130 1134 10.1038/nature03461 15858574

[B30] Balagaddé FK Song H Ozaki J Collins CH Barnet M Arnold FH Quake SR You L A synthetic Escherichia coli predator-prey ecosystem Molecular systems biology 2008 4 187 187 18414488 10.1038/msb.2008.24 PMC2387235

[B31] Danino T Mondragón-Palomino O Tsimring L Hasty J A synchronized quorum of genetic clocks Nature 2010 463 7279 326 330 10.1038/nature08753 20090747 PMC2838179

[B32] Erdmann T Howard M ten Wolde PR Role of spatial averaging in the precision of gene expression patterns Phys Rev Lett 2009 103 25 258101 10.1103/PhysRevLett.103.258101 20366291

[B33] Yu J Xiao J Ren X Lao K Xie XS Probing gene expression in live cells, one protein molecule at a time Science (New York, NY) 2006 311 5767 1600 3 10.1126/science.1119623 16543458

[B34] Boyer M Wisniewski-Dyé F Cell-cell signalling in bacteria: not simply a matter of quorum FEMS microbiology ecology 2009 70 1 19 10.1111/j.1574-6941.2009.00745.x 19689448

[B35] Ravn L Christensen A Molin S Givskov M Gram L Methods for detecting acylated homoserine lactones produced by Gram-negative bacteria and their application in studies of AHL-production kinetics Journal of Microbiological Methods 2001 44 3 239 251 10.1016/S0167-7012(01)00217-2 11240047

[B36] Peccoud J Ycart B Markovian modeling of gene-product synthesis Theoretical Population Biology 1995 48 2 222 234 10.1006/tpbi.1995.1027

[B37] Karmakar R Bose I Graded and binary responses in stochastic gene expression Physical biology 2004 1 3-4 197 204 10.1088/1478-3967/1/4/001 16204839

[B38] Iyer-Biswas S Hayot F Jayaprakash C Stochasticity of gene products from transcriptional pulsing Physical Review E 2009 79 3 1 9 10.1103/PhysRevE.79.031911 19391975

[B39] Horsthemke W Lefever R Noise-induced transitions: theory and applications in physics, chemistry and biology 1984 Berlin Heidelberg: Springer-Verlag

[B40] More MI Finger LD Stryker JL Fuqua C Eberhard A Winans SC Enzymatic Synthesis of a Quorum-Sensing Autoinducer Through Use of Defined Substrates Science 1996 272 5268 1655 1658 10.1126/science.272.5268.1655 8658141

[B41] Parsek MR Val DL Hanzelka BL Cronan JE Greenberg EP Acyl homoserine-lactone quorum-sensing signal generation Proceedings of the National Academy of Sciences of the United States of America 1999 96 8 4360 5 10.1073/pnas.96.8.4360 10200267 PMC16337

[B42] Shahrezaei V Swain PS Analytical distributions for stochastic gene expression Proc Natl Acad Sci USA 2008 105 45 17256 61 10.1073/pnas.0803850105 18988743 PMC2582303

[B43] Gillespie D Exact stochastic simulation of coupled chemical reactions The journal of physical chemistry 1977 81 25 2340 2361 10.1021/j100540a008

[B44] Tsimring LS Volfson D Hasty J Stochastically driven genetic circuits Chaos (Woodbury, NY) 2006 16 2 026103 10.1063/1.2209571 16822035

[B45] McMillen D Kopell N Hasty J Collins J Synchronizing genetic relaxation oscillators by intercell signaling Proceedings of the National Academy of Sciences of the United States of America 2002 99 2 679 10.1073/pnas.022642299 11805323 PMC117365

[B46] Pai A You L Optimal tuning of bacterial sensing potential Molecular systems biology 2009 5 286 286 19584835 10.1038/msb.2009.43 PMC2724973

[B47] Boedicker JQ Vincent ME Ismagilov RF Microfluidic confinement of single cells of bacteria in small volumes initiates high-density behavior of quorum sensing and growth and reveals its variability Angewandte Chemie (International ed in English) 2009 48 32 5908 11 10.1002/anie.200901550 19565587 PMC2748941

[B48] Carnes EC Lopez DM Donegan NP Cheung A Gresham H Timmins GS Brinker CJ Confinement-induced quorum sensing of individual Staphylococcus aureus bacteria Nature chemical biology 2010 6 41 5 10.1038/nchembio.264 19935660 PMC4201857

[B49] Hagen SJ Son M Weiss JT Young JH Bacterium in a box: sensing of quorum and environment by the LuxI/LuxR gene regulatory circuit Journal of Biological Physics 2010 36 3 317 327 10.1007/s10867-010-9186-4 PMC2868974 21629592

[B50] Bennett MR Hasty J Microfluidic devices for measuring gene network dynamics in single cells Nature reviews. Genetics 2009 10 9 628 38 10.1038/nrg2625 19668248 PMC2931582

[B51] Kaufmann G Sartorio R Lee S Rogers C Meijler M Moss J Clapham B Brogan A Dickerson T Janda K Revisiting quorum sensing: discovery of additional chemical and biological functions for 3-oxo-N-acylhomoserine lactones Proceedings of the National Academy of Sciences 2005 102 2 309 10.1073/pnas.0408639102 PMC544315 15623555

[B52] Roberts C Anderson KL Murphy E Projan SJ Mounts W Hurlburt B Smeltzer M Overbeek R Disz T Dunman PM Characterizing the effect of the Staphylococcus aureus virulence factor regulator, SarA, on log-phase mRNA half-lives Journal of bacteriology 2006 188 7 2593 603 10.1128/JB.188.7.2593-2603.2006 16547047 PMC1428411

[B53] Anderson KL Dunman PM Messenger RNA Turnover Processes in Escherichia coli, Bacillus subtilis, and Emerging Studies in Staphylococcus aureus International journal of microbiology 2009 2009 525491 10.1155/2009/525491 19936110 PMC2777011

[B54] Swain P Elowitz M Siggia E Intrinsic and extrinsic contributions to stochasticity in gene expression Proceedings of the National Academy of Sciences 2002 99 20 12795 10.1073/pnas.162041399 PMC130539 12237400

[B55] Pearson J Van Delden C Iglewski B Active efflux and diffusion are involved in transport of Pseudomonas aeruginosa cell-to-cell signals Journal of bacteriology 1999 181 4 1203 9973347 10.1128/jb.181.4.1203-1210.1999 PMC93498

[B56] Xavier K Bassler B Regulation of uptake and processing of the quorum-sensing autoinducer AI-2 in Escherichia coli Journal of bacteriology 2005 187 238 10.1128/JB.187.1.238-248.2005 15601708 PMC538819

[B57] Wang L Hashimoto Y Tsao C Valdes J Bentley W Cyclic AMP (cAMP) and cAMP receptor protein influence both synthesis and uptake of extracellular autoinducer 2 in Escherichia coli Journal of bacteriology 2005 187 6 2066 10.1128/JB.187.6.2066-2076.2005 15743955 PMC1064054

[B58] Herzberg M Kaye I Peti W Wood T YdgG (TqsA) controls biofilm formation in Escherichia coli K-12 through autoinducer 2 transport Journal of bacteriology 2006 188 2 587 10.1128/JB.188.2.587-598.2006 16385049 PMC1347309

[B59] Li J Wang L Hashimoto Y Tsao CY Wood TK Valdes JJ Zafiriou E Bentley WE A stochastic model of Escherichia coli AI-2 quorum signal circuit reveals alternative synthesis pathways Molecular systems biology 2006 2 67 10.1038/msb4100107 17170762 PMC1762088

[B60] Koseska A Zaikin A Kurths J García-Ojalvo J Timing cellular decision making under noise via cell-cell communication PloS one 2009 4 3 e4872 10.1371/journal.pone.0004872 19283068 PMC2652718

[B61] Lu T Shen T Bennett MR Wolynes PG Hasty J Phenotypic variability of growing cellular populations Proceedings of the National Academy of Sciences of the United States of America 2007 104 48 18982 7 10.1073/pnas.0706115104 18025471 PMC2141894

[B62] Shahrezaei V Ollivier J Swain P Colored extrinsic fluctuations and stochastic gene expression Molecular Systems Biology 2008 4 196 10.1038/msb.2008.31 18463620 PMC2424296

